# Multi-professional and multi-agency model PLUSS to facilitate early detection and support of pre-school children with neurodevelopmental difficulties – a model description

**DOI:** 10.1186/s12913-022-07815-8

**Published:** 2022-03-30

**Authors:** Berit M. Gustafsson, Samina Steinwall, Laura Korhonen

**Affiliations:** 1 Department of Psychiatry and Rehabilitation, Psychiatric Clinic, Högland Hospital, Region Jönköping County, Jönköping, Sweden; 2 Department of Child and Adolescent Psychiatry and Department of Biomedical and Clinical Sciences, Linköping University, Linköping, Sweden; 3 Department of Child and Adolescent Psychiatry, Division of Psychiatry and Rehabilitation, Region Jönköping County, Jönköping, Sweden; 4 Barnafrid and Department of Biomedical and Clinical Sciences, Linköping University, Linköping, Sweden

**Keywords:** Preschool child, Neurodevelopment, Child health care, Service development, Intervention, Multi-agency, Multi-professional, Preschool

## Abstract

**Background:**

Neurodevelopmental difficulties, such as problems in social inter-relatedness, communication, motor coordination, and attention, are frequent in preschoolers and constitute a risk for later negative consequences. This article describes the development of a multi-professional and multi-agency model, PLUSS, to facilitate care and interventions for preschoolers with neurodevelopmental difficulties.

**Methods:**

The PLUSS model was developed for children aged 1.5–5 years with a need for a further assessment of neurodevelopmental symptoms. The model is evaluated using a quasi-experimental study design along with qualitative interviews that study preschool teacher, and parent experiences of PLUSS. Outcomes of interest are a) implementation, b) effectiveness related to processes and multi-agency collaboration, c) capacity building among professionals, d) child-related outcomes with a longitudinal follow-up as well as d) parental wellbeing and satisfaction.

**Results:**

The model was launched in 2019 and so far, approximately 130 children have been assessed. Results from a pilot study with 62 children (27–72 months; boys: girls 2.65:1) show that the total mean SDQ score in parental rating was 15 ± 6 and in preschool teacher ratings 14 ± 7, exceeding the Swedish cut-off of 12. 54 parents have participated in parental training and rate high levels of satisfaction (mean score 4.5, max 5.0). In addition, 74 pre-school professionals have been trained in early signs of neurodevelopmental difficulties to facilitate early detection. Feedback from participants indicates high satisfaction with educational activities (mean score 4.2, max 5.0 = very satisfied).

**Conclusions:**

The pilot study shows that the screening procedure can detect children with clinically significant problems. In addition, participant satisfaction is high in parent- and preschool teacher training. The longitudinal study approach enables both child follow-up and evaluation of interventions provided by the working model.

**Trial registration:**

Clinical Trials 2021, PLUSS identifier, NCT04815889. First registration 25/03/2021.

##  Background

Current research shows that due to wide variability of neurodevelopmental symptoms such as problems in social inter-relatedness, communication, motor coordination, and attention, these difficulties can be difficult to detect and diagnose early in life. Also, structural barriers and under-identification of girls and racial/ethnic minorities may delay receiving a timely diagnosis. The earlier the difficulties are present, the higher the probability for several other symptoms [1, 2]. For preschool children, the prevalence of emotional and behavioral problems in international studies varies from 12 to 26% [3–6]. These figures are often substantially higher than those based on diagnostic criteria according to DSM-IV, for example, ADHD 2–8%, 2–7% [7–10], and Oppositional Defiant Disorder (ODD) 2–7%.

C Gillberg [1] has described the concept of ESSENCE (Early Symptomatic Syndromes Eliciting Neurodevelopmental Clinical Examinations) including various difficulties that become apparent during childhood such as language problems, motor skill deficiencies, increased activity level, and impaired function of shared attention. In the Swedish population, the frequency of early (i.e., before school-start) identifiable ESSENCE difficulties is approximately 13% of all boys and 7% of girls. Around half of these children have been examined at a health care clinic by a doctor, speech therapist, or psychologist before school starts. Some of these, but not all, children subsequently meet the criteria for a specific diagnosis.

There is often a great need for support in the close network to the affected child. Despite this, the current health care system rarely provides a multi-professional approach to these preschool children with various difficulties [1]. Even if a diagnosis may be clarifying, efforts to mitigate a child´s day-to-day problems may be the most important for the families. Early developmental difficulties also entail an increased risk of impaired mental health, failure at school, crime, and social exclusion in adulthood [1]. These facts underline the importance of early intervention with optimal support for both strengths and difficulties [11, 12]. Mental health is also strongly affected by demographics, ethnicity, and social factors, which is important to pay attention to in research and clinical work [13–15].

The PLUSS model is based on Bronfenbrenner’s bioecological model. According to this theory, a child´s development is influenced by person-, process-, context- and time-dependent factors in various micro- (e.g., family, and preschool teachers) and macrosystems (e.g., preschool structure and policies) [16]. Also, a dynamic interplay between risk and protective factors play a role in shaping the development over time [17]. Thus, a child´s behavior shows age-related developmental changes [18, 19]. For example, motor skills, language, self-esteem, and emotional regulation are competencies that support a child´s functional ability in everyday life [20–22].

The available research on child development, and the Swedish recommendations from the National Board of Health and Welfare, emphasize the importance of early identification of a need for support and preventive actions. The Swedish child health care (CHC) and also Jönköping County´s work procedures are in line with the National Board of Health and Welfare’s recommendations on early detection of developmental difficulties in children [23]. However, we see a need for further development to fully implement the guidelines and meet the needs of children and their parents [15].

In Sweden, follow-up of a child´s development is done at well-baby clinics by child health care nurses[24]. If needed, further assessment is done in specialized health care. There are specialized psychiatric teams/units for children below school-age (0–6 years) in some parts of Sweden, however not in Jönköping, and the other Nordic countries. These units collaborate with other multi-agency professionals like preschool teachers, who can identify difficulties and promote good mental health [25]. Interestingly, E Fält, A Sarkadi, and H Fabian [26] reported that the quality of CHC routine visits for 3–5-year-olds improved when preschool teachers and parents actively participated as informants.

Several studies have shown that group-based support programs for parents can improve emotional and behavioral problems in preschool children [27]. Also, parental psychoeducation and counseling in communication skills and behavioral modification are important [28]. Early preventive interventions for pre-school children have beneficial long-term effects and are financially motivated [12]. The most effective intervention seems to be parental and pre-school interventions starting at an early age with a focus on communication with the child [29–31].

In this paper, we describe the development of a novel multi-professional and multi-agency model of care, PLUSS (Mental health, learning, development, collaboration around pre-school children), and its adaptations to the catchment area of the county of Jönköping in Southeast of Sweden. The model is designed to match the needs of families and pre-school children with neurodevelopmental difficulties. This “one way in”-model provides coordinated services to screen, assess, and offer different interventions to the target group. In addition, we present initial data to demonstrate how the implementation of the PLUSS model has started.

## Methods

### Design and settings of the PLUSS-model

**Fig. 1:**
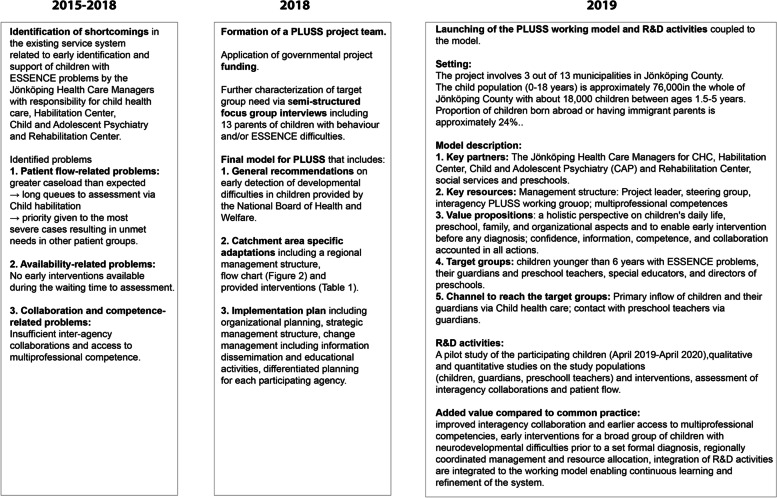
Summary of the development process of the PLUSS model of care

Figure 1 summarizes the PLUSS model and how it was developed. Shortly, since 2015 the CHC in Jönköping has developed routines for early identification of autism spectrum disorders in young children. As part of this work, several service-system-related problems were identified. One central obstacle was patient flow-related difficulties such as caseload that exceeded the system´s capacity. This resulted in long queues of children at the Habilitation Center, which provides services to children with a congenital disability, or a disability acquired early in life and who need habilitation services. Due to the long waiting times, the most severe cases were prioritized at the expense of children with mild to moderate difficulties. Yet another identified problem was the lack of early group-based interventions that could be provided during the waiting time to further assessment for children with suspected autism as well as for a broader group of children with various other neurodevelopmental difficulties (ESSENCE). The third identified major obstacle was insufficient inter-agency collaboration and early access to multi-professional competence.

To tackle these obstacles The Jönköping Health Care Managers for CHC, Habilitation Center, Child and Adolescent Psychiatry (CAP), and Rehabilitation Center (speech therapist) conceived an idea of a regional, interagency team with multi-professional competence (pediatrics, child and adolescent psychiatry, and habilitation, pre-school pedagogy and sociology) that could develop a new working model, the PLUSS, built on the already existing structures provided by the CHC, family centers, preschools, social services and within the specialist level. In addition, the model was expected to follow the general recommendations on early detection of developmental difficulties in children provided by the National Board of Health and Welfare.

To substantiate the understanding of the service system-related problems that the professionals had identified as well as to identify the catchment area-specific needs for adaptations, three semi-structured focus group interviews were conducted as part of the PLUSS development. These interviews included 13 parents of children with behavior and/or ESSENCE difficulties. Parents' experiences of assessment and interventions from Child Health Care, Child and Adolescent Psychiatry, and Child and Youth Habilitation before PLUSS were studied. The qualitative analysis led to four main categories that should be considered in the design of the working model: confidence, information, competence, and collaboration. Participants described a long wait for their child to be assessed and a lack of information that caused feelings of doubt. Furthermore, a lack of competence among health care professionals and a lack of cooperation with the family was described. Parents requested support to help their child whilst waiting for the assessment process. Increased competence for professionals was also asked for in areas of children's mental health and development. The need for improvements could be detected by taking part in parents' experiences with health care and health care professionals.

In 2019, a steering group and an operative multi-professional and multi-agency operative working group ("The PLUSS team") consisting of local, experienced professionals were established. Also, a project leader was assigned to the PLUSS. Apart from the organizational and management structure planning, also other parts of implementation such as change management including continuous information dissemination and educational activities targeted to participating professionals were planned and conducted. In addition, differentiated planning was done for each of the participating agencies.

In summary, the added value of the PLUSS working model compared to common practice in Sweden is improved interagency collaboration and earlier access to multi-professional competencies, early interventions for a broad group of children with neurodevelopmental difficulties before a set formal diagnosis as well as regionally coordinated management and resource allocation. In addition, research and development (R&D) activities are integrated into the working model enabling continuous learning and refinement of the system.

### The patient flow in the PLUSS model

The PLUSS model currently operates in three out of 13 municipalities in Jönköping County, Southeast Sweden with a child population (1.5–5 years) of 76,394 individuals. Approximately 24% of the children are born abroad or have immigrant parents, which is in line with the national average of 25%. Based on local statistics approximately 1,806 children (10%) per year are expected to require assessment for neurodevelopmental difficulties. In the Region Jönköping County, 95% of children in this age group participate in preschool activities and close to 100% attend the CHC routine check-ups. The CHC check-ups are offered free of charge.

**Fig. 2:**
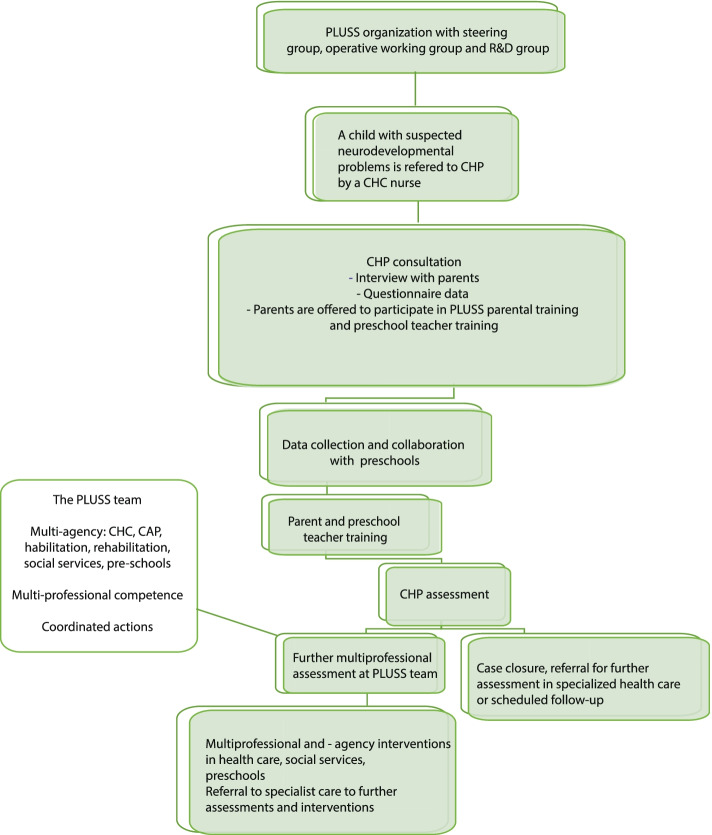
The patient flow in the PLUSS model

The patient flow is summarized in Fig. 2. The PLUSS model is built upon existing processes for patient flow, from early detection to assessment and interventions. No delay of routine practices such as primary assessment or referral is caused to patients in the PLUSS flow. The waiting time for assessment by a child health care psychologist (CHP) is approximately 6 months.

Information about PLUSS is given from a CHC nurse to parents. Parents are then, before the visit to the CHP, asked to fill out questionnaires and sign a consent form for participation in PLUSS. During the first assessment, the CHP interviews the parents and obtains consent for contacting the preschool. The parents are then offered participation in parental training and educational activities (PRIMUS), see below. Data is then collected from the preschool and preschool teachers are offered a training program together with the parents.

The multidisciplinary PLUSS team act as consultants to the CHP to assess the need for further examination within specialist care. This can be done after the completion of interventions or early in the process. After the PLUSS-team conference, parents can join the interventions recommended by the team. The menu of available interventions (Table 1) is a joint living document in which existing, and newly created initiatives, are gathered. The purpose of this list is to make all parties involved in the interventions available, through a clear and structured tool. The intervention list also clarifies the need for coordination between several actors and activities, such as between home and preschool.

**Table 1:** Support provided by the PLUSS

Type of need for support	Type of intervention	Providers	Receiver (C = child, P = parents, G = group, S = staff)
Regulation of emotions and behaviour	Parental support	CHC	P
Problems in social interaction and play with peers	Special pedagogical support supervision and efforts based on organization, group and individual	Pre-school	C, G, S
Speech and language related problems	Parental Education: Series Talks and Social Stories The Swedish National Agency for Education's reading lift, module several languages in the preschool Material that speech therapist/special education teacher will share with preschool staff who work with it from a group perspective	Habilitation	P
Late motor development	Physiotherapist intervention and training	Habilitation	C, P, S
Anxiety	Parental support Support and treatment Preschool: special pedagogical supervision and interventions based on organization, group and individual	CHC, CAP	P, S
Early life adversity	Parental support Support and treatment TMO (trauma-informed care)	CHC, CAP, pre-schools	C, P, S
Attachment problems	WWW Parental support COS-P Attachment trauma – differential diagnosis autism COS-P as individual support Parental support/Family Treatment	CHC, Social Services (SS)	C, P
Suspected or confirmed disability of the child's parent	Following aid decisions: - Parental support/Family Treatment - Collaboration with e.g. LSS or other actors - Network Meetings ) - Contact family - Family home placement/network placement	SS	P
Suspected or confirmed mental illness in the child's family or immediate family	Parental support Adult psychiatry Parental support/Family Treatment Collaboration with e.g. LSS or other actors Network Meetings Contact family Family home placement/network placement	CHC, SS	P
Parental abuse	Parental support/Family Treatment Buy external outpatient care for children and/or adults Network Meetings Contact family Family home placement/network placement treatment via placement or outpatient care for adult drug users	SS	P

The participants of the PLUSS team are required to have a mandate to directly accept referrals to their departments, to reduce the risk that some children "fall between chairs". The time required for each child included in PLUSS is estimated to be 5.5 h, from the CHC nurse information to the PLUSS-team case discussion.

### Parental training PRIMUS

The PRIMUS intervention comprises a total of five lessons, three hours each. It is a manual-based group program for parents with children between the ages of 3–6 years. The lessons are suitable for both small groups (8–12 participants) and large groups (15–30 participants). Lessons are held recurrently at the same time once a week. The topics that are covered in the program include child development, difficulties in concentration, motor skills, perception, tics and compulsions, difficulties in speech and language, social interaction, theoretical thinking as well as daily routines. This intervention aims to increase understanding of the child's difficulties; tools to help the child develop and increase functioning; more harmonious interaction between child and parents; reduced stress and frustration due to conflicts in the family and others around the child, as well as information to parents on where to turn for professional help.

### Training for preschool teachers, special educators, and directors of preschools

Apart from PRIMUS, an intervention for preschool teachers is offered, and Internet-based teacher training which parents also are encouraged to take part in. This intervention aims to facilitate cooperation between parents and preschool, to create a common ground for information sharing and reflection upon different perspectives, in the best interest of the child. The teacher training consists of seven film modules about normal development and common neurodevelopmental difficulties including self-regulation. In addition, communication, the structure of everyday life, and the role of clarifying methods in pedagogy are included. This is further deepened in a workshop, to which parents and preschool teachers are invited. During this workshop, the parents and preschool teachers jointly acquire practical approaches in areas included in the education/training, clarifying methods in pedagogy, communication, and support in everyday life. With the child in mind, the parents and preschool teachers discuss and try different relevant exercises. The participants collect their thoughts in an "action list" in which common strategies and approaches are planned to stimulate the child's development both at home and in the educational environment.

### Other interventions

The available interventions can be individual assessments and/or interventions for the child, family, and preschool (Table 1). These can be offered by a special educator, speech therapist, or occupational therapist; targeted parent groups in the form of "Emotional children"; targeted parent groups in the form of "Toilet school"; support in basic interaction; parental support and treatment; recommendations regarding the social services' general and targeted efforts—in consultation with the social secretary on-site; recommendations regarding the preschool's general and targeted efforts—in consultation with the special educator's competence in the area.

### Data collection

To evaluate the PLUSS model of the care, the child-related outcomes are studied in a quasi-experimental, quantitative study with a longitudinal follow-up. Registration was done in Clinical Trials 2021; PLUSS identifier, NCT04815889. The study subjects are children who are referred to a CHP due to problems related to neurodevelopment. The aim is to collect data on 700 children, which allows analyses of primary outcomes related to the Strengths and Difficulties (SDQ) instrument as well as subgroup analysis and person-based analysis (cluster analysis and/or path analysis) with statistical strength of 0.80 and α < 0.05. Power estimation is based on previous studies by Rothenberger, et al. [24].

Child data is collected using the following instruments: Strength and Difficulties Questionnaire (SDQ) [32, 33] rated by preschool teachers and parents, Child Engagement Questionnaire (CEQ) [34] rated by preschool teachers, LAPS [35] rated by child health care psychologists at baseline and CHC nurses at follow-up and JA-OBS [36] rated by CHC nurses. Data is be collected at baseline, after any PLUSS intervention, and at the regular 5-year old visit to the CHC.

Parent data is collected through a separate questionnaire that covers different background factors such as profession, educational level, mother tongue, or any earlier or present diagnoses. In addition, data on parental stress, the satisfaction of interventions, etc. is be collected. Qualitative data is collected through focus group interviews with parents of children participating in PLUSS.

Structured follow-up research on collaboration between different PLUSS activities is collected with a method called the Spider [37]. This was done before the PLUSS model and then again every 6 months with professionals in the organization working with children/families in the target group.

Register data is used for waiting time follow-up, the number of visits and involved professionals, offered and completed interventions as well as other process-related outcomes. Data collection is managed by researchers and people in the operative working group with a special assignment for follow-up and organization of submitted forms. Data is obtained from parents, preschool teachers, CHC nurses, and CHC psychologists.

A control group (*n* = 160) is recruited from municipalities that have not yet been included in the PLUSS clinical trial. Through this, children/families are not withheld from treatment that they would otherwise have received.

### The pilot study

The PLUSS model pilot study targeted children who were referred to a CHP due to problems related to neurodevelopment during the period of April 2019-April 2020. The difficulties were most often detected at a routine visit to the CHC when the child is 2.5 years old. During this routine visit, a nurse assessed development and screened for developmental difficulties according to the national guidelines with supplemental data collection with CEQ, LAPS, and JA-OBS as part of the PLUSS model of care and its scientific evaluation. The most common causes for a psychologist consultation (and criteria for inclusion in the PLUSS model) were a developmental delay, impairment in social interaction or motor skills, language and communication difficulties, hyperactivity, difficulties with concentration, self-regulation, and behavioral problems, fear, anxiety, or other difficulties in everyday life. Feedback from the child's preschool was collected as a part of routine visits to CHC. No exclusion criteria were applied, except for parents only requesting support in their parental role. Furthermore, there was no eligibility requirement regarding an established diagnosis.

Child and parental data were collected as stated above and included socioeconomic background data, SDQ rated by preschool teachers and parents, CEQ rated by preschool teachers, LAPS rated by child health care psychologists, and JA-OBS rated by CHC nurses. The data was analyzed using SPSS and contained descriptive statistics on demographic data (presented in numbers and percentages). Mean values were analyzed concerning cut-off for SDQ full-scale sum and subscales. Spearman correlation analyses were done between LAPS full-scale sum, SDQ full-scale sum, and between CEQ average and SDQ full-scale sum.

## Results

A pilot study was conducted in the PLUSS project; 62 children (45 boys and 17 girls) aged 27–72 months were included. The mean age was 4.6 years (54 months) with a standard deviation of 12 months. 75.4% of the children had Swedish as their mother tongue and 24.6% had another mother tongue. For demographic data (see Table 2) regarding parents' highest level of education: 6.8% have completed Swedish compulsory school of 9 years, 30.5% upper secondary school, 54.2% higher education 54.2% and 8.5% other education. Regarding employment, 1.8% of the parents were unemployed, 87.3% were employed and 10.9% had other employment (on sick leave). These results correspond well to the country of Sweden as a whole.

**Table 2:** Demographic data including gender, age, mother tongue and information about parents’ education and employment

	**Number**	**Percent**
Girls	17	27.5
Boys	45	72.5
Mean Age (month)	54	
**Children's Mother Tongue**		
Swedish	46	75.4
Other	15	24.6
**Parents´ education level**		
Compulsory school (9 years)	4	6.8
Upper secondary school	18	30.5
Higher education	32	54.2
Other education	5	8.5
**Parents´ employment**		
Unemployed	1	1.8
Employed	48	87.3
Other employment (sick leave)	6	10.9

### Neurodevelopmental problems

In the pilot study, the SDQ estimates from both parents (15.5) and preschool teachers (14.5) captured children in need of support based on the Swedish cut-off value of 12 points for the SDQ full scale and supplements that measure everyday functioning (cut-off 1 point) [2]. The reported scores were above the cut-off for the subscales Hyperactivity and Prosocial Behavior, for both parents and preschool teachers as informants. For the subscales Peer Problems and Emotional Problems, both parents' and preschool teachers' scores were below the cut-off. Furthermore, parents' scores were borderline lower than cut-off values for the subscale Behavioral problems. See Table 3.

**Table 3:** Mean values ​​and cut-off values for SDQ subscales filled out by guardians and preschool teachers. The Mann Whitney U-test (two-tailed) was used for comparisons between boys and girls

	**Swedish cut-off**	**Parent rating**	**Group comparison**	**Preschool** **rating**	**Group comparison**
		mean (SD)	p	mean (SD)	p
**Total score**	12	15 (6)	.058	14 (7)	
Behavioural Problems	4	4 (2)		3 (2)	
Hyperactivity		6 (3)	.046	6 (3)	
Peer problems	4	3 (2)	.034	3 (2.5)	.009
Emotional problems	3	3 (2)		2 (2)	
Prosocial behaviour	3	6 (2)	.014	5 (3)	
**Supplement function**	1	6 (4)	.050	5.5 (3.5)	

Preschool teachers rated boys with more difficulties (*p*. = 0.058, mean 15 ± 6) than girls (mean 11 ± 8) on the total SDQ scale and on the subscales hyperactivity (p 0.046, mean 6.5 ± 3) (girls; mean 5 ± 3), and peer problems (p 0.034, mean 3 ± 2) (girls; mean 2 ± 2.5). Boys were also rated to have significantly more problems in everyday functioning (p 0.050) using the SDQ Supplement (mean 6 ± 3.5) than girls (mean 4 ± 3.5). Boys were also rated to have significantly (p 0.014) less pro-social skills (mean 4 ± 3) than girls (mean 7 ± 3). When parents estimated SDQ, the only significant difference was more peer problems in boys (p 0.009, mean 3 ± 2) than girls (mean 2 ± 2).

When assessing both median and mean values for SDQ subscales, supplements, and full-scale sums, there was no statistically significant difference between how parents and preschool teachers reported the same child, except for the subscale Prosocial Behavior where parents reported higher average values. The pilot study showed that both parents’ and preschool teachers’ estimates are relevant for capturing early symptoms of mental illness in preschool children and that the children included in PLUSS need support.

Spearman correlation analyses showed a positive relationship between LAPS full-scale sum (filled out by CHP) and SDQ full-scale filled out by parents (0.36 rho Spearman, sig 0.01). No correlation was seen between LAPS and SDQ full-scale filled out by preschool teachers. Thus, parents and CHC psychologists tended to evaluate the child similarly. CEQ average score for the children in PLUSS was 2.77 and for the Swedish normal population average score was 3.21 (BM Gustafsson [11]. There was a negative correlation between CEQ average and SDQ full scale filled out by preschool teachers (-0.50 rho Spearman, sig 0.01). Thus children who were seen by preschool teachers as engaged (high values of CEQ), were also estimated to have lower scores in SDQ.

### Parental training

Parents (*n* = 97, divided into 8 groups) of 64 children participated in parental training groups with high levels of satisfaction (satisfaction mean score 4.5, max 5.0). Both parents participated in the group with a proportion of 54%, the only mother 38%, only father 8% and appointed guardians 2%.

### Preschool teacher training

Preschool teachers, special educators, and directors of preschools (*n* = 86, divided into 8 groups) of 54 children were trained in early signs of neurodevelopmental difficulties to facilitate early detection. Feedback from these educational activities is encouraging (satisfaction mean score 4.2, max 5.0).

## Discussion

The PLUSS model has been launched and successfully implemented in the County of Jönköping. Professionals have easily been recruited to the project and have contributed to the development of the processes/interventions included in PLUSS—both within the Region and the municipality's activities.

Based on Bronfenbrenner's theory [16] PLUSS holds an advantage in including social services in the model—allowing the child's entire context to be supported based on the needs of the child. The preschool can also be supported by the interventions supplied by the PLUSS model regarding the child's everyday functioning [38]. The results from the focus groups show that the PLUSS model bridges a gap for parents of children in the target group: early interventions and increased knowledge on neurodevelopmental difficulties were called for, both in health care professionals as well as within preschools. The results from the parents' and preschool teachers' estimates, show high scores of behavioral problems in SDQ and low engagement in CEQ, implying that the target group of PLUSS are children in need of early detection and interventions. This finding is reassuring and indicates that chosen instruments and data collection strategies work as expected. Further evaluations on these issues will be conducted later.

Results from both parents' and preschool teachers' evaluations of the training programs in PLUSS show high satisfaction mean scores (4.5 out of 5) from parents and preschool teachers (4.2 out of 5). Parental training PRIMUS provides an arena for parents to meet and share experiences of children with neurodevelopmental difficulties, and an arena for the preschool and parents to meet and discuss the strengths and difficulties of the child in focus. Further research will address in more detail the effect of the PRIMUS intervention on children´s problems and parental experiences.

Results from previous studies with a similar approach have shown that the most effective intervention seems to be parent and preschool interventions starting at an early age, with a focus on communication [29, 31, 39]. Several studies have shown that group-based support programs for parents can improve emotional and behavioral problems in preschool children [27]. Unlike earlier studies, such interventions will be possible to evaluate long-term within the PLUSS model.

Fewer referrals than expected have been received by the PLUSS team. From January to April 2021, 10 children were referred to the team. One possible factor for causing a delay of referrals is the consent from parents which is obtained on paper, while the CHP assessment interview is held online due to the COVID-19 pandemic, causing a risk for the consent form to be forgotten or delayed. The health care data system in the Region currently neither provides a solution for questionnaires nor for consent forms to be sent in digitally. The results obtained in the pilot study remain to be validated in post-pandemic studies.

The CHP might also find it less time-consuming to refer a child directly to specialist care, than to the PLUSS team, with the concept of further discussing the patient with the team. Although with the intent of an atmosphere of openness, there is a risk for the CHP to feel questioned or criticized during the case presentation, thus increasing the risk for refraining from referral. There might also be a risk that information and understanding of the PLUSS model may not be the same within the whole CHP group—leading to fewer referrals to the PLUSS team. Continuous information about the PLUSS model is sent to the child health psychologists in the Region.

Cooperation between the region and municipality has increased through the PLUSS model. From this, the understanding of the respective activities is expected to increase, leading to beneficial development, and further bridging of identified gaps in the care for preschool children with neurodevelopmental difficulties. The PLUSS-model enables evidence-based methods for behavioral problems, such as the Incredible Years parent program [40] to be included in research work after implementation in a unit such as the CAP in Jönköping.

An important aim for future research in the PLUSS-model is to follow the included children long-term regarding symptom development, established diagnoses, and evaluate given interventions. Parental stress and perceived competence in parents to children with neurodevelopmental difficulties are also important to study.

To improve the mental health of children in Sweden, a suggestion of five evidence-based interventions was recently published, one of them being early detection and early interventions for young people with risk for future mental health problems [41]. Current knowledge indicates that early detection and early interventions for children are beneficial long-term and economically justifiable [12, 42]. The PLUSS model has been constructed to better meet the demands of health care today with early interventions to parents and children exhibiting neurodevelopmental difficulties. However, the model is designed in the context of one Region in Sweden and thus generalization and transfer of the model to other environments should be addressed in separate later studies.

To summarize, the article describes the development of a PLUSS model of care used currently in the County of Jönköping in Sweden. The model is developed to tackle identifies obstacles in the care taken of children with neurodevelopmental difficulties and has involved multiple agencies and parents. The model and included interventions will be studied further using a quasi-experimental study design.

## Conclusions

The PLUSS model includes early screening and early detection of neurodevelopmental difficulties in preschool children, and group interventions are offered before any established diagnoses. The model facilitates collaboration between health care and municipal activities. Through the PLUSS screening procedure with SDQ questionnaires, children in need of support can be detected. The preliminary results show that both parents and preschool teachers’ estimates are relevant for capturing early symptoms of mental illness in preschool children. Encouraging results are seen in parent and preschool evaluations of given interventions.

## Data Availability

The datasets used and/or analyzed during the current study are available from the corresponding author on reasonable request.

## Abbreviations

ADHD: Attention deficit hyperactivity disorder
CAP: Child and adolescent psychiatry
CEQ: Child engagement questionnaire
CD: Conduct disorder
CHC: Child health care center
CHP: Child health care psychologist
ODD: Oppositional defiant disorder
GAD: Generalized anxiety disorder
JA-OBS: Joint attention observation tool
LAPS: *Lapsen psykososiaalisen terveyden arviointimenetelmä (child mental health assessment form)*
PLUSS: Psykisk hälsa Lärande Utveckling Samverkan kring Små barn (mental health, learning, development, collaboration around pre-school children)
SAD: Separation anxiety disorder
SDQ: Strengths and difficulties questionnaire
